# Influenza Neuraminidase Characteristics and Potential as a Vaccine Target

**DOI:** 10.3389/fimmu.2021.786617

**Published:** 2021-11-16

**Authors:** Sarah Creytens, Mirte N. Pascha, Marlies Ballegeer, Xavier Saelens, Cornelis A. M. de Haan

**Affiliations:** ^1^ Vlaams Instituut voor Biotechnologie (VIB)-UGent Center for Medical Biotechnology, VIB, Ghent, Belgium; ^2^ Department of Biochemistry and Microbiology, Ghent University, Ghent, Belgium; ^3^ Section Virology, Division Infectious Diseases & Immunology, Department of Biomolecular Health Sciences, Utrecht University, Utrecht, Netherlands

**Keywords:** influenza, neuraminidase, antigenic drift, monoclonal antibodies, correlate of protection, vaccines

## Abstract

Neuraminidase of influenza A and B viruses plays a critical role in the virus life cycle and is an important target of the host immune system. Here, we highlight the current understanding of influenza neuraminidase structure, function, antigenicity, immunogenicity, and immune protective potential. Neuraminidase inhibiting antibodies have been recognized as correlates of protection against disease caused by natural or experimental influenza A virus infection in humans. In the past years, we have witnessed an increasing interest in the use of influenza neuraminidase to improve the protective potential of currently used influenza vaccines. A number of well-characterized influenza neuraminidase-specific monoclonal antibodies have been described recently, most of which can protect in experimental challenge models by inhibiting the neuraminidase activity or by Fc receptor-dependent mechanisms. The relative instability of the neuraminidase poses a challenge for protein-based antigen design. We critically review the different solutions that have been proposed to solve this problem, ranging from the inclusion of stabilizing heterologous tetramerizing zippers to the introduction of inter-protomer stabilizing mutations. Computationally engineered neuraminidase antigens have been generated that offer broad, within subtype protection in animal challenge models. We also provide an overview of modern vaccine technology platforms that are compatible with the induction of robust neuraminidase-specific immune responses. In the near future, we will likely see the implementation of influenza vaccines that confront the influenza virus with a double punch: targeting both the hemagglutinin and the neuraminidase.

## 1 Introduction

Influenza A and B viruses (IAV and IBV) cause acute respiratory illness and are widespread in the human population, with seasonal appearance in moderate climate zones and year-round manifestation in the tropics (1, 2). The use of licensed influenza vaccines is considered one of the best measures to prevent human influenza. These vaccines are vital in the efforts to alleviate the burden of influenza illness and deaths and are especially recommended for individuals who have an increased risk of developing complications due to age or underlying disease (3, 4). The effectiveness of currently licensed influenza vaccines however leaves considerable room for improvement. Depending on the IAV subtype and the antigenic match between the influenza strains that are represented in the vaccine and the strains that circulate in the population, the vaccines prevent 10 to 60% of laboratory-confirmed medically attended influenza (5). The composition of seasonal influenza vaccines is reconsidered every year for each hemisphere in an attempt to keep pace with the antigenic drift of the viral hemagglutinin (HA), the major envelope protein on the influenza virions and the principal protective antigen in currently used influenza vaccines. These annual updates come with a risk of suboptimal predictions leading to a mismatch between the vaccine- and circulating influenza virus strains. There is a pressing need for more effective influenza vaccines that can elicit stronger and potentially broader protection against influenza. In the past decade, there has been a renewed interest in the exploration of influenza neuraminidase (NA) as a protective antigen component in influenza vaccines. Here, we review some of the seminal findings on NA structure and function, its immune-protective potential, as well as the current efforts to implement NA in next-generation influenza vaccines that aim for eliciting an immune response with increased magnitude and breadth.

## 2 Neuraminidase: Structure and Function

### 2.1 NA Structure

**Figure 1:**
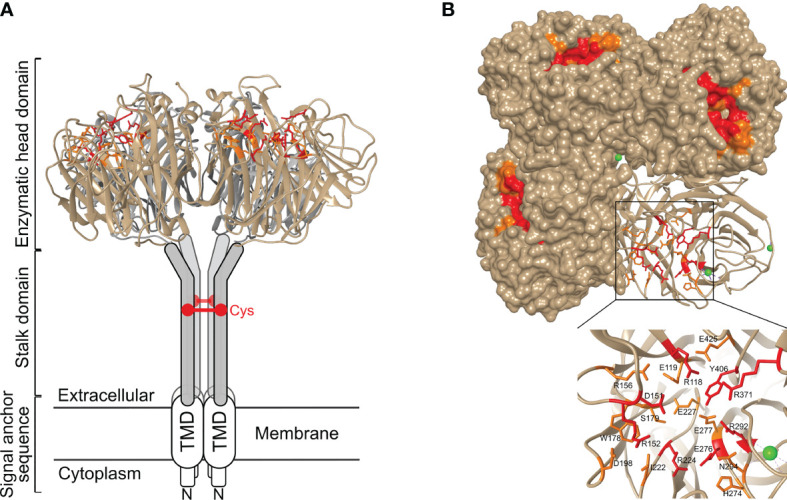
Structure of neuraminidase and its catalytic site. **(A)** Side view and **(B)** top view of N1 NA (PDB 6Q23). NA is a homotetrameric type II membrane protein consisting of a head domain, stalk domain, and a transmembrane domain (TMD) and cytoplasmic tail that together form the signal anchor sequence. In general, NA stalk domains contain a cysteine residue (Cys) involved in intermolecular disulphide bond formation. The inset in panel **(B)** shows the catalytic site with the residues that interact with the sialic acid-containing substrate depicted in red, and the residues that stabilize the catalytic site labelled in orange. Ca^2+^ ions are shown as green spheres.

NA is one of the three membrane proteins expressed on IAV and IBV particles, next to HA and matrix protein 2 (M2). Label-free protein quantification of purified influenza A and B virions revealed that the NA : HA ratio ranges from 0.1 to 0.2 (6). NA is a homotetrameric type II membrane protein with a mushroom-like shape. Each protomer comprises approximately 470 amino acid residues and consists of a cytoplasmic tail, a transmembrane domain (TMD), a stalk and a head domain (**Figure 1**).

The cytoplasmic tail of NA consists of 7 highly conserved amino acid residues. Alanine-scanning mutagenesis of the cytoplasmic tail of NA of A/WSN/33 virus indicated a role in virus budding. Notably bud release rather than bud formation was affected in the tested NA cytoplasmic tail mutants (7). The cytoplasmic tail is also important for the association of NA with lipid rafts. Lipid rafts on the apical site of polarized cells are sites where newly formed influenza virions initiate assembly and budding (8). Together with the cytoplasmic tail, the TMD plays a role in the transport of newly expressed NA to the apical plasma membrane (9). The sequence of the TMD is moderately conserved across IAV subtypes and predicted to form an alpha helix. The TMD ensures the membrane anchoring of NA and serves as a translocation signal. In addition, this domain is reported to be an important stabilizing factor for the tetrameric NA formation (10, 11). Substitution of hydrophilic residues in the TMD by alanine reduced or even abolished the interaction between the four TMDs of the NA tetramer (10).

The stalk domain connects the TMD with the catalytic head domain. There is no crystal structure available of the stalk domain. The NA stalk varies in length within and across NA subtypes, carries multiple predicted N-glycosylation sites and, with few exceptions, contains at least one cysteine residue that can form an intermolecular disulphide bond with a neighbouring NA molecule (12). Glycosylation of the stalk region may contribute to NA stability, whereas inter-stalk disulphide bond formation is important for the tetramer formation. Whereas the length of the NA stalk of human influenza viruses seems to be relatively constant, the NA stalk of avian NA subtypes, in particular N1, N2, N3, N5, N6, and N7, tolerate deletions (13). The stalk length affects NA enzymatic activity, presumably by modulating the accessibility to the sialic acid-containing substrates (14).

Crystal structures of the catalytic head domain of at least one representative NA from N1 to N9 and from influenza B NA have been resolved (15–22). The NA head domain is characterized by a six-bladed propeller that is folded around the catalytic site and which is typical for all known sialidases (23). Each blade is made up of four antiparallel β-sheets that are stabilized by disulphide bonds and connected by loops of variable length (12). Each monomer harbours a catalytic site, oriented towards the lateral side of the NA head, that is highly conserved over the different IAV subtypes (**Figure 1**). Remarkably, unlike the tetramer, influenza NA monomers and dimers show very little if any enzymatic activity (24). It is not known why tetramer formation is essential for NA to be active. Possibly, this is linked to calcium binding by tetrameric NA, which contributes to NA activity and stability. NA can bind up to nine Ca2^+^ ions in the case of the 2009 H1N1 pandemic (H1N1pdm09) virus derived NA (16, 25–28).

Despite the significant primary sequence variation between IAV subtype NAs, the catalytic site residues in N1–N9 NAs are highly conserved. Among these, residues R118, D151, R152, R224, E276, R292, R371, and Y406 (N2 numbering) directly contact the substrate, while residues E119, R156, W178, S179, D198, I222, E227, H274, E277, N294, and E425 play a key role in stabilizing the catalytic site residues (19) (**Figure 1**). Phylogenetic analysis indicates that IAV NAs fall into two distinct groups. In group 1 NAs, a cavity adjacent to the catalytic site is observed which is absent in group 2 NAs (29). This cavity is created by the so-called 150-loop that consists of residue 147-152 and is flexible in group 1 NAs such that it can adopt an open and a closed conformation. Group 2 NAs on the other hand lack this second cavity as the formation of a salt bridge between D147 and H150 stabilizes the 150-loop, which is absent in group 1 NAs due to the presence of a G147 there (29, 30). The catalytic site and its adjacent 150-cavity are further explored as targets for NA inhibitors (31). Additionally, most avian influenza NAs, but not NAs of human viruses, have a functional second sialic acid binding site (2SBS) or hemadsorption site next to the catalytic site (32). The 2SBS consists of three loops with residues that facilitate binding to sialic acid in its so-called chair conformation. The catalytic site, in contrast, binds sialic acid in its twisted boat conformation (16). Studies comparing human and avian NA catalytic properties show that the presence of a functional 2SBS in avian NA increases NA activity against multivalent substrates (33, 34).

### 2.2 NA Function

**Figure 2:**
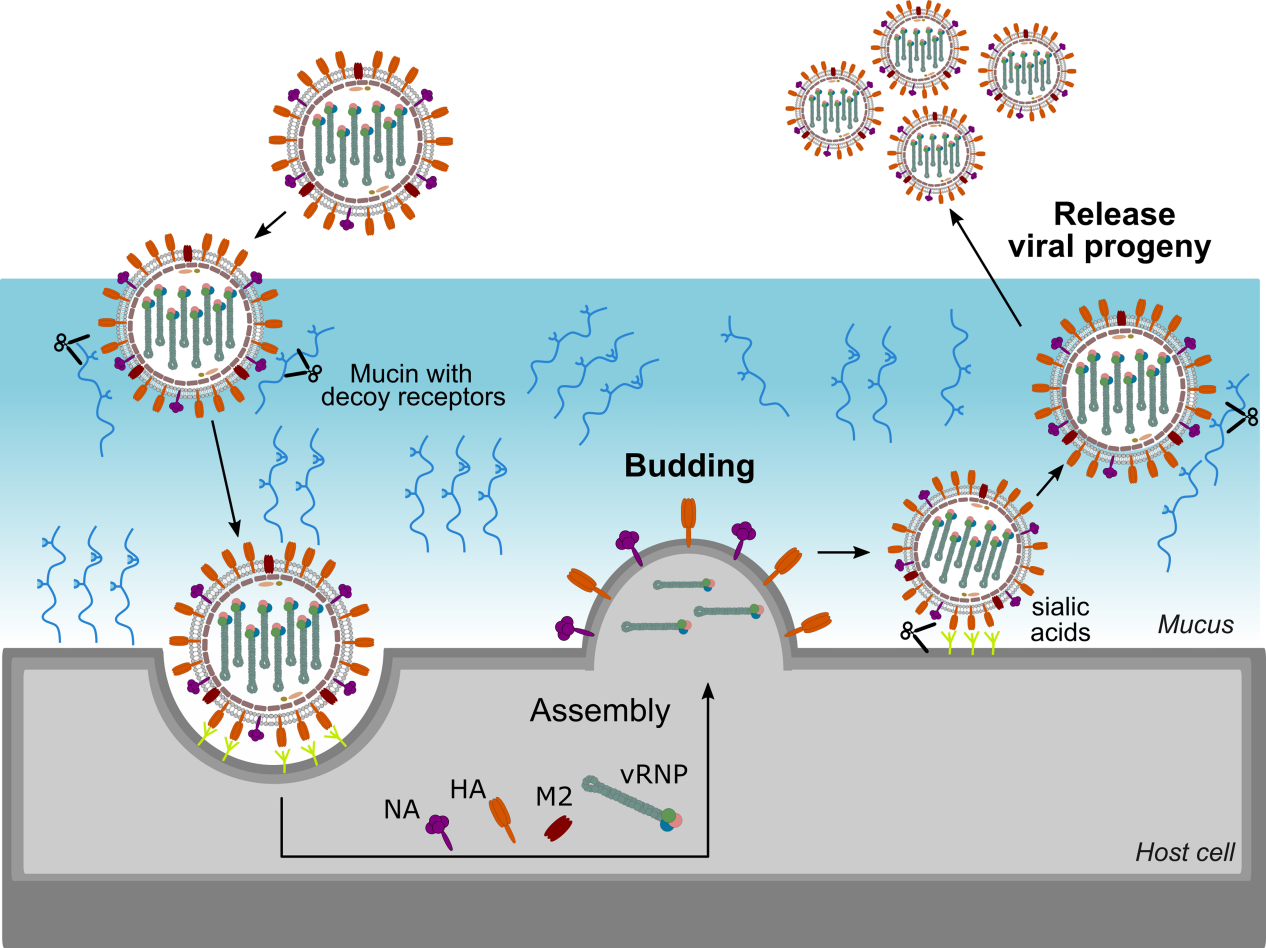
Important role for NA in the virus life cycle. NA contributes to virus motility, allowing the virus to move through the mucus layer and to reach functional receptors at the cell surface. NA also plays an essential role at the end of the virus life cycle by removing sialic acids from the cell surface thereby allowing efficient release of virions and preventing virion aggregation.

HA and NA exert different functions in the influenza virus life cycle. HA is vital in the entry process, by mediating binding to sialic acids on host cell glycoproteins or -lipids, which results in virion uptake into endocytic vesicles, and the subsequent fusion of the host cell and virus membrane through a pH-induced conformational change (35). NA, on the other hand, catalyses the removal of the terminal sialic acids and thus functions as a receptor-destroying enzyme. NA activity is involved in multiple steps of the virus life cycle (**Figure 2**). During viral entry NA cleaves decoy receptors present in the mucus that lines the epithelial cells of the respiratory tract, allowing the infection of underlying epithelial cells (36–38). In line herewith, inhibition of NA activity was shown to result in severely decreased infection of differentiated primary human airway epithelium cells (39). NA activity was also reported to stimulate HA-mediated membrane fusion (40). The best-known function of NA in the influenza virus replication cycle is its critical role in the release of newly formed virions from the infected cell and in prevention of HA-mediated virion aggregation by removing sialic acid from the viral and host cell membrane (41).

Given these partly opposing functions, a functional balance between HA and NA is critical for viral replication. If HA binding is much stronger than NA activity, for example, virions may be trapped by decoy receptors present in mucus and eventually be removed by mucociliary clearance before reaching the underlying epithelial cells. On the contrary, if NA activity dominates, the virion will quickly move through the mucus, but will more likely fail to stably bind to an epithelial cell and enter it. HA and NA can be found on the virion surface in a patch-wise distribution, which contributes together with the HA-NA balance to virus motility and entry. In filamentous influenza virions, NA is mainly localized at one pole of the filamentous virus, and the few NA molecules present along the side of the virus tend to cluster in patches as well (42). Such polarized viruses seem to move by Brownian ratchet-like diffusion in a mucus-rich extracellular environment, in which filamentous particles exhibit directed mobility away from their NA-rich pole and toward the HA crowded part of the virus (42). HA binding to sialic acid is reversible due to the low affinity. This reversible binding likely allows NA patches to catalyse the removal of the sialic acid when the HA is no longer bound thereby initiating movement of the virion through mucus and over the cell surface. This NA-driven virion motility presumably allows the virus to find and to dock to specific spots on the cell surface that trigger entry into the host cell (38, 43, 44). Although detailed mechanistic insights into the importance of the HA-NA balance are still lacking, it is clear that besides HA also NA contributes to IAV pathogenicity. For example, truncation of the NA stalk, which has been associated with adaptation of IAV from aquatic birds to poultry, resulted in increased virulence in poultry and mice (13, 45–49).

## 3 Antibodies Directed Against NA as a Correlate of Protection

Protection against disease caused by influenza virus infection has traditionally been correlated with the presence of hemagglutination-inhibiting (HAI) antibodies in the blood. Such antibodies can neutralize influenza viruses *in vitro*, by preventing the binding of the virus to its sialic acid-containing receptor on the target cell. Therefore, in order to induce such antibodies, current influenza vaccines are standardized for their HA content. Natural infection, however, elicits an immune response against both HA and NA (50). In mice it has been shown that NA inhibiting (NAI) antibodies are associated with a reduction in pulmonary virus titer. More recently, by using a guinea pig model, monoclonal NAI antibodies were demonstrated to reduce airborne transmission of human IAVs, both when the antibodies were administered post infection to the infected animals or to the exposed recipients (51). This observation is in line with the contribution of NA to the release and spread of newly formed viruses after infection (50, 52, 53). Further, a combination of HA and NA provides even enhanced protection against influenza compared to HA alone (54–57). Studies comparing protection in mice induced by conventional inactivated influenza vaccines with or without supplementation with recombinant NA showed that for protection against homosubtypic influenza virus infection anti-HA antibodies sufficed. However, when challenged with an influenza virus with a mismatched HA supplementation of the vaccine with NA was required to reach a clear reduction in pulmonary virus titer (58, 59). In a ferret study it was found that whereas vaccination with HA reduced viral titers, vaccination with NA particularly decreased the clinical effects of infection, with optimal protection being achieved by a combination of the two antigens (54).

Evidence of NA-based protection in human has also been observed during the 1968 Hong-Kong pandemic. This pandemic was caused by a H3N2 virus with the same N2 as the previously H2N2 circulating virus. Individuals with pre-existing antibodies against the NA of H2N2 were less likely to be infected with the newly emerged virus (60–63). In humans, NA-inhibiting antibodies in serum correlate with a reduced virus load in nasal wash (62). Couch et al. first showed that serum N2-specific antibodies, elicited by vaccination with H1N2 (H1 HA from A/equine/Prague, N2 NA from A/Aichi/2/68 (H3N2)) in humans who initially lacked anti-HA antibodies, correlate with a reduction of viral shedding after challenge of the subjects with A/Aichi/2/68 (H3N2) virus (62). Three decades later, during the 2009 H1N1 pandemic season, it was demonstrated that HAI and NAI antibodies in serum independently correlated with immunity against infection and infection-associated illness. Moreover, in H1N1pdm09-infected individuals, NAI antibodies in serum independently predicted reduced illness (64). This correlation of NAI antibodies with protection is also supported by more recent studies in humans. The presence of NAI antibodies in serum was associated with a reduction of PCR-confirmed influenza infection for both H3N2 and H1N1pdm09 virus (65, 66). Memoli et al. divided the participants in their controlled human challenge study with H1N1pdm09 virus in a group based on HAI or NAI titer and showed that subjects with a high (> 1:40) NAI titer at baseline not only presented with reduced symptoms and virus shedding duration, but also with a reduction in the number of symptoms and the symptom severity. In contrast, HAI titers correlated only with a reduction in the number of symptoms and virus shedding duration but not with symptom severity (66). These results highlight that anti-NA immunity can enhance protection against influenza virus infection.

## 4 Evolution and Antigenic Drift of NA

HA and NA are prime targets of the host’s immune response to influenza virus infection (67). Combined with the relatively high mutation rate of the replicating influenza virus RNA genome, human influenza viruses with antigenically altered HA and NA emerge that have a selective advantage over previously circulating strains because they are less likely to be recognized by antibodies that prevail in the population. This phenomenon of antigenic changes is called antigenic drift, and is at the basis for the frequent updating of human influenza vaccines. The selection of seasonal vaccine strains is based on three types of data: epidemiological information, HA and NA gene sequence phylogeny and serological analysis using an HA inhibition assay. Today, the main focus of genetic and antigenic surveillance is thus on HA since licensed influenza vaccine formulations are standardized for the amount of HA (68). The antigenic drift of HA has been extensively studied (69). In a seminal paper, Smith et al., discerned a pattern of eleven antigenic HA clusters in human H3N2 viruses that circulated between 1968 and 2003 (70). Later, two clusters were added to the antigenic map when HAI data was added up to 2011 showing that the human H3N2 virus continued to evolve antigenically (71). Interestingly, the antigenic change between the HA clusters is the result of a limited set of amino acid changes confined to positions near the receptor binding site of HA (72, 73). In contrast to HA, antigenic drift of NA is not routinely examined. In the next paragraphs, we discuss the genetic evolution of NA and the limited studies on NA antigenic drift.

### 4.1 Genetic Evolution

**Figure 3:**
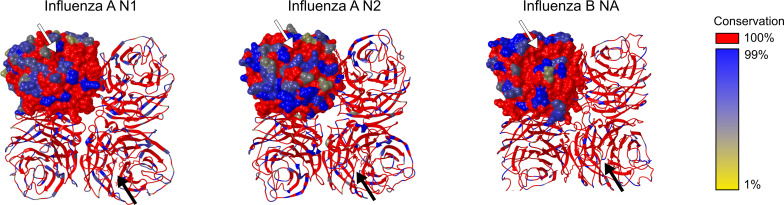
NA structural conservation. Structural alignments of all seasonal influenza vaccine included influenza strains in the period 1970-2021.The sequence conservation of Influenza A N1, Influenza A N2 and Influenza B NA amino acid residues was visualized using MUSCLE and shown on a crystal structure of the respective NA subtype using the render by conservation function in CHIMERA. Residues conserved in all sequences of a specific subtype NA are shown in red. Further distinction of conservation is indicated from dark blue (99% conservation) till yellow (1% conservation). Structure representations based on N1: PDB 4B7Q, N2: PDB 4GZX, and IBV NA: PDB 4CPL. Arrows point towards the catalytic site.

Several studies addressed the genetic evolution of HA and NA using IAV and IBV strains, which revealed that their evolution differs and is often asynchronous (74–78). Although the general topology of the NA and HA phylogenetic trees is similar with the typical ‘ladder-like’ gradual evolution and rapid replacement of old strains by newer ones, NA evolved slower and more gradually at the nucleotide level than HA. Looking at 40 years of evolution of human H3N2 viruses, Westgeest et al. showed that NA had fewer nucleotide substitutions over this time span compared with the HA head HA1, the most variable part of the HA gene (78). Nevertheless, it was concluded by Bhatt et al. (74) that with almost all adaptive evolution in NA being concentrated in residues on the surface of NA, the adaptation rate is higher for surface NA residues than for HA1. The observation that adaptive evolution in NA occurs almost exclusively in solvent-accessible surface residues indicates an important role for antibody-mediated immune responses in NA evolution. In **Figure 3**, the IAV N1, IAV N2 and IBV NA structural conservation for the viruses that were used in seasonal influenza vaccines from 1970 to 2021 are depicted. Overall, a relatively high conservation of the head domain residues is observed for each NA (sub)type. As expected, the catalytic site is for all NA subtypes highly conserved. However, the surface residues do show variation, especially for IAV N2, which is in agreement with a higher rate of adaptation for NA in H3N2 than H1N1 viruses (74).

### 4.2 Antigenic Drift

The analysis of NA antigenic drift usually relies on the determination of NAI titers of polyclonal ferret antisera raised against a particular influenza virus strain (e.g. a vaccine strain) in an enzyme-linked lectin assay (ELLA) (79, 80). The antigenic relatedness of the NAs from two viruses can be assessed by comparing NAI titers, calculating percent relatedness (81) or by performing antigenic cartography (76) to generate an antigenic map (80). On such a map, based on the quantification of the raw data of NAI assays, the antigenic distances between influenza viruses or sera are visualized. Importantly, the reactivity of antisera of ferrets that have experienced a single experimental virus infection is quite different from human sera which have an antibody repertoire that has typically been shaped by multiple prior infections and vaccinations (80, 82). The antigenic changes identified this way based on sera from ferret that were infected once with a particular influenza virus strain may therefore not always accurately reflect the response of an (adult) human individual (83) although such discrepancies have so far only been demonstrated for HA-specific responses (84–86).

Only a few studies have investigated the antigenic drift of NA using large NAI data sets and by constructing antigenic maps. In 2011, Sandbulte et al. reported on the characterization of the antigenic drift of NA in human H1N1 and H3N2 viruses that were recommended for influenza vaccine implementation over a period of 15 years in the USA (76). Similarly, Gao et al. examined the antigenic drift of NA sequences of H1N1pdm09 viruses (80). Overall, the antigenic differences between NAs of human H1N1 viruses occurred in one direction, meaning that antisera raised against older strains reacted weakly with more recent NAs whereas reactivity between antisera against more recent strains and older NAs was high (76, 80, 87). Both raw NAI data and the antigenic map obtained by Sandbulte et al. show that the NA of pre-2009 seasonal human H1N1 was in antigenic stasis for over a decade despite its genetic evolution during that time. For human H3N2 viruses the antigenic drift of NA was also not proportional to the number of amino acid changes induced. Collectively, these data show that there is discordance between the antigenic and genetic evolution of NA. Indeed, For N2 it was found that a single point mutation (E329K) was responsible for the abrupt NA antigenic drift between 2006 and 2007 (76). Similarly, for H1N1pdm09 few substitutions appeared to be largely responsible for the observed antigenic changes (80). The presumed important role in antigenic drift of K432E (80) was later confirmed using recombinant NA proteins that only differed at this position (87). Epistatic interactions as well as by biophysical constraints may also play a role in NA antigenic drift as was shown for the evolution of an antigenic site in H3N2 NA (88, 89). Furthermore, changes in HA receptor binding, e.g. as a result of antigenic drift, may in turn select for substitutions in NA that affect enzymatic activity to restore a functional balance in HA and NA, while additionally affecting NA antigenicity (90).

Bidirectional IAV transmission between humans and swine represents a serious public health challenge. In the last decade, an increasing number of zoonotic infections with IAV from swine has been reported. For example, after two distinct human-to-swine H3N2 spill overs in 1998 and 2002, N2-98 and N2-02 lineage viruses have circulated in swine in the USA and antigenically evolved over the next 20 years. After IAV infection, pigs typically produce broadly cross-reactive NAI antibodies to the N2 protein, but no NAI cross-reactivity between the N2-98 and N2-02 lineages was observed (91). The antigenic distance between swine and human N2 antigens increased over time to the extent that there is by now very little antigenic similarity with the human seasonal H3N2 NA that these viruses were derived from, nor with the NA of currently circulating human H3N2 viruses. This suggest that there will be little to no cross-reactive NA mediated immunity in both the swine and human population which may impact the occurrence of future spill overs (91).

## 5 Neuraminidase-Specific Monoclonal Antibodies: Mechanisms of Action and Epitopes

Using large panels of monoclonal antibodies (mAbs) or recombinant NA proteins, several studies have set out to identify antigenic sites on NA (12, 52, 92–96). NA activity and inhibition thereof can be evaluated with two types of assays. The so-called 4-methylumbelli-feryl N-acetyl-α-D-neuraminic acid (MUNANA, a fluorogenic NA substrate) or NA-STAR (a chemiluminescent NA substrate) assays allow to quantify the hydrolysis of a small soluble substrate by NA. The small molecule NA inhibitor oseltamivir, which binds precisely inside the catalytic site, inhibits NA activity in these types of assays (97). Only a mAb that binds inside or in very close proximity to the catalytic site will be able to prevent cleavage of the small substrate whereas mAbs that bind distal to the catalytic site are less likely to have an effect in such assays.

**Figure 4:**
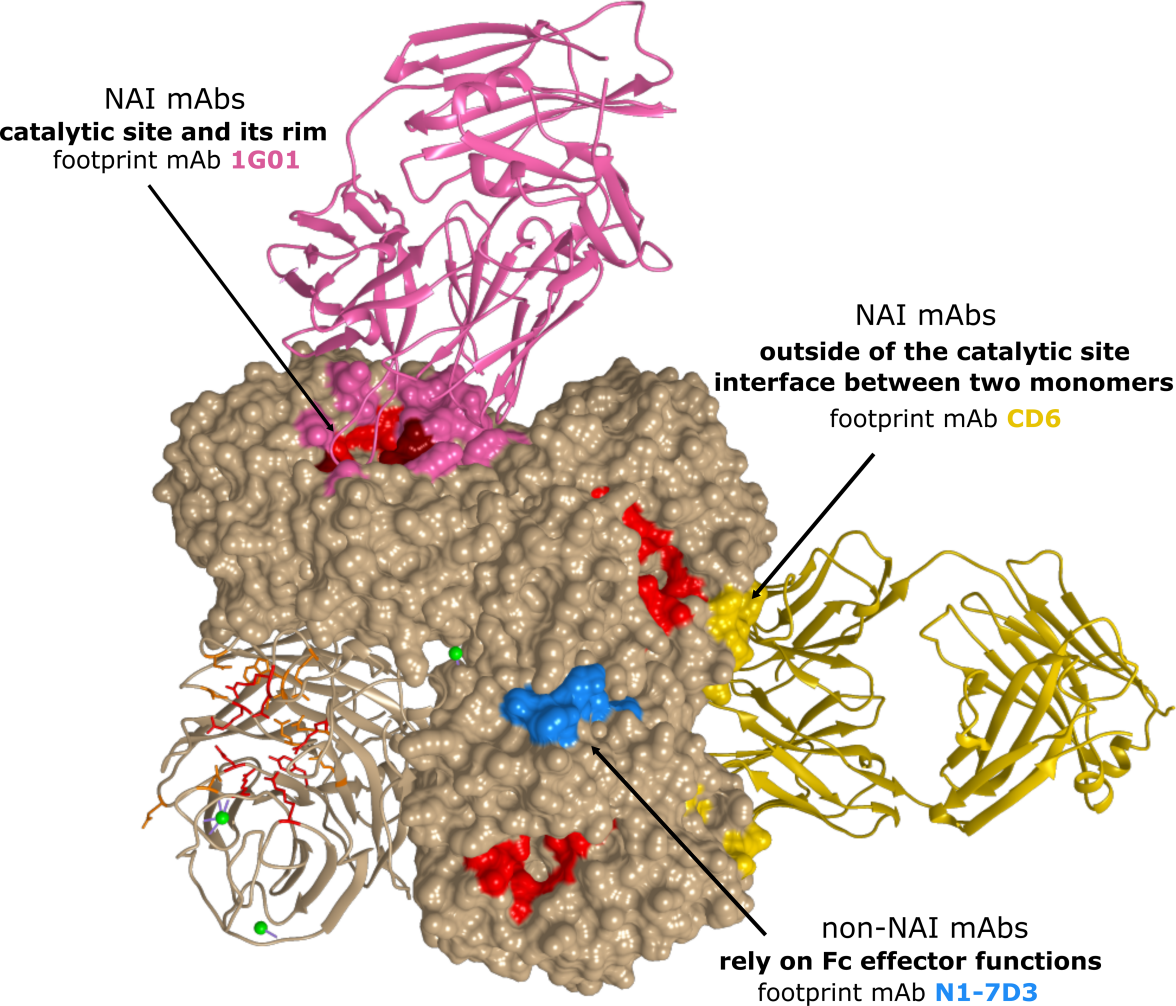
Antigenic regions of N1 NA-specific mAbs. The A/California/04/2009 (H1N1) tetramer is depicted with one protomer in ribbon representation. The catalytic residues are depicted in red. For each region, a representative antibody footprint is shown: 1G01 (105) in pink (PDB 6Q23) with antibody contact residues in NA that overlap with the catalytic site in dark red; CD6 (103) in gold (PDB 4QNP) and N1-7D3 (113) in light blue.

ELLA assays use larger substrates compared to MUNANA and NA-STAR assays, such as the glycoprotein fetuin that contains sialylated N- and O-linked glycans. A mAb binding to the NA head domain, even outside of the catalytic site, may still sterically block access of fetuin to the catalytic site and prevent cleavage of the substrate, while cleavage of small soluble substrates may not be affected. Comparing the inhibition profile of NAI mAbs based on the outcome of both types of assays will thus give an indication on their possible binding site. **Table 1** provides an overview of different NA-specific mAbs and their impact on NA activity as determined with a small molecule substrate or in the ELLA. Overall, two different antigenic regions have been described that characterize NAI antibodies: (i) the catalytic site and its rim and (ii) outside of the catalytic site, including the interface between two adjacent monomers. Additionally, several NA-specific mAbs have been described that lack detectable NAI activity in either assay, yet can protect *in vivo* against influenza A virus challenge. These mAbs rely on Fc effector functions and will be discussed separately (**Figure 4**).

**Table 1:** Overview of NAI mAbs and their epitopes.

Epitope	Mechanism of action	Neuraminidase inhibition?		mAb
				(reference)
				* structural data available Bold: mentioned in text
		Small substrate assay	Large substrate assay	
The catalytic site and its rim	(partially) block access to catalytic site	Yes	Yes	NA-73* (98, 99)
				NA-108 (98, 99)
				HCA-2 (100, 101)
				IG05 (102)
				2E01 (102)
				**HF5** (103, 104)
				3G1 (52)
				229-1 F06 (50)
				229-1G03 (50)
				229-11)05 (50)
	CDR3 loop insertion in catalytic site	Yes	Yes	IG01* (105)
				IG04* (105)
				IE01* (105)
				Z2B3* (106, 107)
				NA-45* (98, 99)
	bind loop surrounding the cavity of the catalytic site	Yes	Yes	Mem5* (108)
				NC10* (109)
				NC41* (110)
				IG8* (111)
				229-1D05 (50, 112)
				229-1C02 (50, 112)
Outside of the catalytic site	bind linear epitope on tip	No	Yes	NA-63* (98, 99)
				NA-80* (98, 99)
	unknown	No	Yes	IF2 (52)
				N1-C4 (113)
	bind interface between adjacent monomers causing steric hindrance	No	Yes	CD6* (103)
				**N8-4** (114)
				4F11 (52)
				NA-22* (98, 99)

Neuraminidase inhibition activity is determined via small substrate (MUNANA or NA-STAR) and large substrate (ELLA) assays. mAbs in bold are mentioned in the text, while other mAbs are referenced for completeness.

### 5.1 NAI mAbs That Target the Catalytic Site and Its Rim

Early antibody characterization and vaccination studies have shown that immunity to NA can be very broad within, but usually not across, subtypes (105). Nevertheless, mAbs that directly bind into the highly conserved catalytic site of NA (115) and inhibit enzymatic activity in small molecule assays are expected to exert a degree of heterosubtypic NAI. Recently, Stadlbauer et al. reported on a human mAb, 1G01, which binds to and inhibits the activity of several group 1 and group 2 NAs as well as NAs from both influenza B virus antigenic lineages *in vitro*. MAb 1G01 was shown to inhibit NA activity in a NA-Star assay and to occupy the catalytic site *via* a long CDR H3 loop by co-crystal structure analysis (**Figure 4**) (105). A similar mechanism of action was described for mAb NA-45 where the CDR H3 loop adopts a protruding conformation with a tip that inserts into the NA catalytic site (98). This type of substrate mimicry is unique among all structurally characterized NA antibodies so far but has also been reported for anti-HA antibodies that target the receptor-binding site (116, 117). Interestingly, the number of residues in the antibody footprint might be important for the cross-reactivity of mAbs. For example, the binding footprint of 1G01 (105) and Z2B3 (106, 107) significantly overlaps. However, the 1G01 footprint includes more catalytic and framework residues which explains the broader cross-reactivity of 1G01 compared to Z2B3 (107). It is important to note that the footprint of mAbs that bind into the highly conserved catalytic site of NA often overlaps with the rim of the catalytic site. The rim is less conserved and can tolerate amino acid substitution without NA losing enzymatic activity.

Next to conventional mAbs, other antibody moieties that target the NA catalytic site have been described. The first NA-specific single domain antibodies or VHHs were described by Harmsen et al. with several candidate cross-NA binders and some VHHs that could affect NA activity (118). The isolation and characterization of a set of alpaca-derived H5N1 NA-specific VHHs (N1-VHH) with NA-inhibitory activity was also described. Two monovalent candidates N1-3-VHHm and N1-5-VHHm could inhibit NA activity with N1-3-VHHm also inhibiting oseltamivir resistant H5N1 virus NA. Bivalency of these constructs enhanced their NA inhibitory capacities and resulted in VHH constructs that could protect mice against H5N1 challenge (119).

### 5.2 mAbs that Bind to Epitopes Outside the Catalytic Site

A large proportion of the reported NAI mAbs only display NAI activity in the ELLA, but not in assays with small molecule substrates, indicating they do not contact the catalytic site directly (99). Binding of these mAbs likely results in steric hindrance and restricts access of large glycoconjugate substrates to the catalytic site (52). The NAI activity in ELLA assays has been associated with the capacity to block virus egress from infected cells (98, 99). An example of such a mAb is CD6, which effectively inhibited H1N1pdm09 viruses both *in vivo* and *in vitro* (103). Interestingly, CD6 more efficiently inhibited NA as an intact IgG compared to Fab and Fab_2_ molecules of CD6, demonstrating that CD6 inhibits NA activity through steric hindrance and not structural distortion. Crystallization studies revealed a quaternary epitope that spans the lateral faces of two neighbouring N1 monomers (**Figure 4**). Such a quaternary epitope present in intact NA dimers had been proposed earlier by Saito et al. for the N8-4 mAb but until then had never been structurally defined (114). For HA, quaternary epitope-specific mAbs that span two HA monomers that are efficient in neutralizing virus *in vitro* have also been described (120, 121). Some studies showed a correlation between the relative distance of epitopes to the NA catalytic site and the *in vitro* properties of mAbs. For example, mAb HF5 which has contact residues that surround the catalytic site pocket, showed more efficient NAI activity compared to other mAbs with an epitope that is located more laterally on the NA head (92, 107). Additionally, murine anti-N9 mAbs that inhibit NA enzymatic activity with a small molecule substrate *in vitro*, provided superior *in vivo* protection compared with mAbs that inhibited NA activity only in the ELLA assay (93).

### 5.3 mAbs That Depend on Fc Effector Functions for Protection

It is becoming increasingly clear that Fc-mediated effector functions have an important role in providing *in vivo* protection against influenza virus challenge (122). The fragment crystallisable (Fc) region of an antibody can interact with Fc receptors and thereby mediate indirect effector functions such as antibody-dependent cellular cytotoxicity (ADCC), antibody-dependent cellular phagocytosis (ADCP) and complement-dependent cytotoxicity (CDC) (123). Using Fc receptor knockout mice and DA265 mutant mAbs that are unable to bind Fc receptors, it was demonstrated that broadly reactive HA-stalk antibodies depend on Fc-Fcγ receptor interactions *in vivo* (122, 124, 125). The contribution of Fcγ receptor engagement for *in vivo* protection by some NA-specific mAbs was demonstrated by Job et al. (126). The mouse monoclonal antibody N1-7D3 binds to a conserved linear epitope near the carboxy-terminus of N1 NA and shows no NAI activity (**Figure 4**) (113). However, a recombinant mouse-human chimeric version comprised of the variable domains of mouse N1-7D3 and the constant regions of human IgG1 could engage activating Fcγ receptors and protected FcγR-humanized mice against challenge with H1N1pdm09 virus (126). In addition, mAbs with no detectable NAI activity reported by Yasuhara et al., could protect mice against a lethal influenza virus infection. Interestingly, a N297Q mutant version of these mAbs that lacks Fcγ receptor-binding activity failed to protect, thereby supporting the crucial role for Fcγ effector functions in protection by non-NAI mAbs (127). In line with this, grafting of the non-NAI N1-7-VHH on a mouse IgG2a Fc could protect mice against an otherwise lethal H5N1 challenge (119).

Several studies have demonstrated that the Fc-mediated effector functions might also be crucial for *in vivo* protection by some anti-NA mAbs that possess weak NAI activity (99, 127). Thus, while for potent N9 NAI mAbs Fc effector functions were not needed, the ability to interact with Fc receptors was required for weakly NAI mAbs to protect against challenge (99). Likewise, a broadly cross-reactive mAb with low NAI activity required FcγR interactions to mediate protection, while a strain-specific mAbs with high NAI did not (124). Likely, the sum of the NAI activity and the FcγR-mediated effector functions determines the potency of an anti-NA antibody *in vivo*. An anti-NA mAb with high NAI activity can reduce virus spread *in vivo* mainly through its NAI activity, whereas an anti-NA mAb without NAI activity can suppress virus replication mainly through FcγR-mediated effector cell activation (127).

## 6 NA-Based Influenza Vaccines

### 6.1 NA Immunogenicity in Seasonal Influenza Vaccines

Despite the recognized importance of NA-reactive antibodies in protection against influenza, the current commercially available influenza vaccines do not consistently elicit these antibodies (50, 128). NA immunogenicity of these vaccines varies widely between manufacturers (65, 128–130) and is poor compared to natural infection. Whereas following natural infection the number of NA-reactive B cells were equal to or exceeded HA-reactive B cells, current vaccines rarely induced NA-reactive B cells (50). Furthermore, broadly cross-reactive NAI antibodies elicited by natural infection were unable to bind multiple commercially available inactivated vaccines, indicating that the vaccines lack the NA epitopes targeted by these antibodies (50).

The poor NA immunogenicity of the licensed influenza vaccines can be attributed to a few factors. As mentioned, current vaccines are optimized and standardized specifically for inducing high HAI antibody titers. As a result, the immunogenicity of the NA is not guaranteed. Quantity and quality of the NA antigen varies between manufacturers and vaccine batches (131, 132). Virus inactivation procedures may also affect NA immunogenicity. While treatment with EDTA or formalin did not compromise the immunogenicity of recombinant NA proteins (133), native NA in a viral particle may respond differently to these conditions. The stability and immunogenicity of NA also varies between strains (132). Nevertheless, in case vaccination does elicit NA antibodies, these can be long lived in healthy human subjects (134, 135). Better consideration of the NA component of current vaccines could therefore mean an important step forward in effectivity of the current vaccines. This could be achieved by also considering NA in selection of the vaccine strains, optimizing the manufacturing process to keep NA antigenically intact, and also standardizing the amounts of immunogenic NA in vaccine preparations.

### 6.2 Next-Generation NA-Based Vaccines

**Figure 5:**
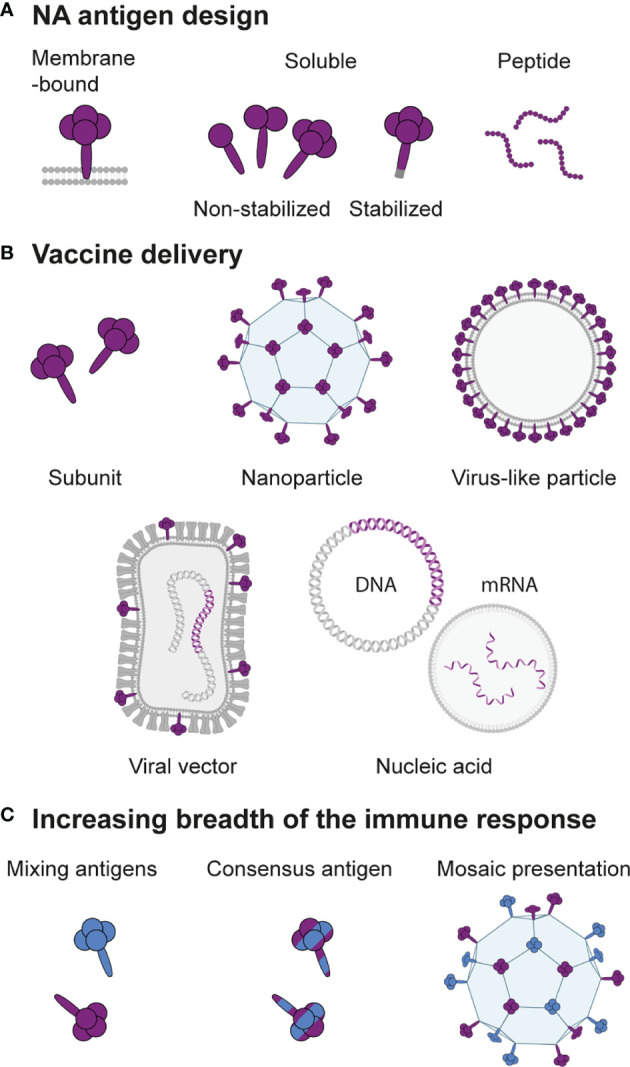
Design of next-generation NA-based vaccines. **(A)** The NA antigen can be presented in the native membrane-bound form, as a soluble protein that lacks the transmembrane domain with or without modifications to retain or stabilize the tetrameric structure, or as peptides containing an epitope of interest. **(B)** Depending on the antigen design, various methods for vaccine delivery are possible. Soluble NA can be administered as a subunit vaccine or coupled to a nanoparticle carrier. Membrane-bound NA can be presented on a virus-like particle or on the cell surface when encoded as RNA or DNA delivered directly or by a viral vector. **(C)** Strategies to increase the breadth of the immune response include mixing of NAs from different strains, computational design of consensus NAs, or hetero-multivalent mosaic presentation of NAs from different strains on a single particle.

Next-generation seasonal and universal influenza vaccines call for an improved NA-directed immune response. Various options for rational design of the NA antigen and mode of presentation to the immune system are in development aiming to boost the breadth and magnitude of the NA-specific response (**Figure 5**). The following section summarizes promising ideas and the rationale behind them.

#### 6.2.1 Enhancing NA Immunogenicity Within Existing Influenza Virus Vaccines

In addition to optimizing the manufacturing conditions of the current seasonal vaccines, extra steps can be taken to improve the immunogenicity of NA while using the same influenza virion-based vaccine technology as a basis. For example, by re-structuring parts of the viral genome, a higher amount of NA can be incorporated into the viral particles (136, 137). Swapping the packaging signals of HA and NA that contain the viral RNA promoters resulted in virions with more NA, although at the expense of HA. Immunization with this virus resulted in increased levels of NAI and Fc-dependent antibodies directed against NA and induced protection against lethal challenge with an NA-matched IAV strain. HA-directed IgG titers were however significantly lower for the recombinant virus compared to the wild-type (136). Since glycosylation of the NA head domain was also found to affect NA incorporation in the virion (138), altering glycosylation sites could be explored as an alternative strategy to incorporate higher levels of NA in an IAV vaccine strain. Such modifications may, however, also affect NA antigenicity and immunogenicity. Extending the NA stalk so that NA surpassed HA in length was also shown to enhance NA immunogenicity of an inactivated virion-based vaccine in mice. Specifically, increased levels of antibodies with ADCC activity were induced. The authors hypothesized that their extended NA stalk domain method exposes NA epitopes to the immune system that were otherwise hidden (139).

#### 6.2.2 Recombinant Protein Design

An alternative solution to increase the immune response to NA is to add extra NA to vaccine formulations. Johansson et al. supplemented inactivated vaccines with soluble NA purified from viral particles. This approach induced higher titers of NAI antibodies without compromising on HAI antibodies (58, 59). Recombinant soluble NA proteins (**Figure 5A**) are also attractive vaccine antigens to supplement inactivated virus vaccines or as a component of a multi-antigen subunit vaccine. Important in the design of such antigens is that a correctly folded tetrameric structure is essential for optimal immunogenicity (133, 140). Often enzymatic activity is measured as a proxy for a correct conformation of the NA antigen. While this is a reliable proxy for the presence of intact antigenic structure, NA activity in itself is not required to induce protective NA-specific antibodies in mice (133, 141, 142). Early on, recombinant NA was produced by replacing the membrane anchor sequence with a signal sequence derived from a type I membrane protein. This method typically results in a mix of tetrameric, dimeric, and some monomeric protein (24). To produce pure and stable tetrameric NA, ectodomains are better fused to heterologous tetramerization domains. Multiple tetramerization domains have successfully been used to produce immune-protective recombinant NA: VASP, GCN4, and Tetrabrachion (54, 143, 144). Tetrabrachion-stabilized NA proteins appear most stable and enzymatically active, presumably due to the parallel orientation of these domains (145, 146). In addition, a crystal structure of a tetrabrachion-stabilized N9 showed that the head domain maintained its native structure as found on virions (147). Whether immunization with these constructs also results in better quality antibody responses is yet to be determined. Interestingly, a recent study showed that the combined choice of tetramerization domain and inclusion of the stalk domain into recombinant NA profoundly impacts activity and immunogenicity. Thorough evaluation of individual recombinant NAs may therefore be required to determine the optimal antigen design strategy (142).

Mutations in the NA stalk (148) or the interface between protomers (149) can also enhance the stability and immunogenicity of recombinant NA. Cysteine mutations in the stalk led to more efficient dimer formation of recombinant N1, resulting in enhanced enzymatic activity and immune protection. The cysteine-stabilized dimers were however still outperformed by a VASP-stabilized tetrameric NA (148). It was only recently recognized that recombinant NA proteins can adopt an open conformation in addition to the closed conformation that is found on the surface of influenza virions. Structure-guided stabilization of the closed conformation was performed that improved thermal stability. More importantly, it also enhanced the affinity to protective antibodies elicited by viral infection, indicating that these antigens may elicit antibody responses to vulnerable quaternary epitopes more efficiently (149).

To broaden the protection elicited by a recombinant protein antigen, computational methods can be used to design constructs based on a consensus sequence of varying strains (**Figure 5C**). After similar techniques have shown promise for the induction of more broadly reactive HA responses (150), recent studies engineered NA constructs that combined sequences of various human, swine, and avian H1N1 and H5N1 strains (151, 152). The resulting antigens were immunogenic and induced antibodies against a broader range of viruses than wild-type NA antigens, including NAI antibodies against strains not included in the antigen design (151). Immunization or passive transfer of the sera of immunized mice offered protection against homologous and heterologous viral challenge (151, 152). While the broadened immune response may come at the cost of a lower magnitude compared to immunizations with a matched NA antigen (152), consensus NA antigens are promising candidates for inclusion in multi-antigen universal vaccine formulations.

#### 6.2.3 Multivalent Presentation

The immunogenicity of soluble protein antigens can be substantially improved with multivalent presentation. Multivalent nanoparticles better mimic viruses in terms of size, shape, antigen valency, and repetitive organization, resulting in enhanced uptake by antigen presenting cells and stronger activation of B cell receptors (153). Such nanoparticle designs may constitute virus-like particles (VLPs) containing membrane-anchored NAs or protein nanoparticles to which soluble NAs are coupled (**Figure 5B**).

NA can self-assemble into VLPs, either when expressed alone or in combination with other IAV structural proteins (154–157). VLPs that display N1 alone or in combination with H5 and M2e induced high titers of NAI antibodies (154, 157–159). Vaccination with N1 VLPs derived from H1N1pdm09 provided cross-protection against lethal challenge with heterologous (H5N1) and even heterosubtypic (H3N2) IAV (159). However, in a more recent study N1 VLPs were not able to cross-protect against a historical H3N2 strain (160). N2 VLPs derived from a more recent H3N2 virus did protect against challenge with the distant H3N2. In addition, bivalent vaccination combining the N1 and N2 VLPs induced strong anti-N1 and -N2 antibody responses that exceeded the antibody levels induced by either one of the VLPs individually (160). Protective efficacy of NA-based VLP vaccination has also been demonstrated in ferrets. In these animals, VLPs containing H5N1-derived NA and matrix protein M1 induced high serum NAI antibody titers and protection against lethal homologous challenge. Incorporation of additional H5 or H3 into the VLPs further reduced clinical symptoms of the ferrets after H5N1 challenge. VLPs composed of H3/N2/M1 were however unable to cross-protect against the heterosubtypic H5N1 challenge (154). Efforts to further boost both humoral and cellular immunity of VLP vaccines currently focuses on attachment of adjuvants directly onto the VLPs (161–163).

In a different strategy based on multivalent presentation, nanoparticles consisting of little more than IAV structural proteins were generated (164). A dense core was constructed out of M2e protein and decorated with M2e-NA fusion proteins by chemical cross-linking. These double-layered nanoparticles greatly improved immunogenicity and (cross-)protection over soluble M2e-NA fusion proteins. M2e-NA nanoparticles were generated using NA from H5N1 and H3N2. Mice immunized with the M2e-N1 nanoparticles were fully protected against mortality following challenge with the homologous strain, as well as H1N1 and H3N2. Immunization with the M2e-N2 nanoparticles fully protected against mortality following challenge with the homologous strain and an H9N2 virus and conferred 60% protection against H1N1 (164).

Self-assembling protein nanoparticles are highly ordered, monodisperse carrier platforms that are increasingly used in experimental vaccines. The geometry of these nanoparticles is versatile and can be readily adapted for optimization to a specific antigen (165). While these carriers have not yet been used to generate NA-based nanoparticle vaccines, examples of their application for other viral glycoproteins illustrate their promise. HA nanoparticles, for example, elicited more than 10-fold higher antibody titers compared to the commercial inactivated vaccine, including antibodies directed against conserved vulnerable epitopes (166). The respiratory syncytial virus fusion protein similarly induced 10-fold higher neutralizing antibody titers when presented on a two-component protein scaffold (167). Self-assembling protein scaffolds are also well suited for presenting viral glycoproteins from multiple strains together on mosaic nanoparticles (**Figure 5C**). It was hypothesized that presentation of these diverse antigens alongside each other gives an avidity benefit for cross-reactive B cell receptors, resulting in a broader antibody response. This strategy was applied using the HA receptor binding domains (RBDs) of two H1N1 strains. Mosaic nanoparticles displaying two distinct H1 RBDs were found to induce broader antibody responses than a mixture of homotypic nanoparticles displaying the same set of RBDs (168). These results indicate that mosaic nanoparticles may enhance activation of B cells specific for the otherwise subdominant cross-reactive epitopes, which could be an interesting strategy to evaluate for NA.

#### 6.2.4 Epitope-Based Vaccines

Epitope-based vaccine design aims to precisely direct the immune response towards conserved vulnerable B or T cell epitopes by presenting peptide epitopes (**Figure 5A**) on an immunogenic carrier or in a multi-epitope construct. In doing so, these techniques have potential for eliciting broadly protective immunity. The potential of an universally conserved linear B cell epitope near the NA catalytic site consisting of residues 222 through 228 or 230 (NA_222_) as an vaccine antigen was recently studied (169, 170). Kim et al. incorporated the epitope into the HA head domain of a H1N1 virus, creating a chimeric virus that was inactivated prior to immunizations. The NA_222_ chimeric virus, but not the inactivated wild-type virus, induced a strong Th1-type antibody response directed to this epitope. The chimeric virus protected against heterosubtypic challenge with H3N2 and H9N2 viruses and at 6 days post-challenge the mice expressed high levels of mucosal IgG and IgA specific for the epitope (169). Zeigler et al. developed a self-assembling protein nanoparticle containing the NA_222_ epitope in addition to two universal CD4 T cell epitopes that mediate high-affinity, long-lived antibody responses. The NA_222_ nanoparticle induced high IgG titers and conferred approximately 50% survival in otherwise lethal H1N1 and IBV challenge, whereas nanoparticles with HA or M2e epitopes were 70-75% protective with similar IgG titers. It was suggested that the NA_222_ epitope was less protective due to limited antibody accessibility (170). Alternative novel B and T cell epitopes may be identified using *in silico* predictions and be combined into a multi-epitope construct. Further *in vivo* studies are needed, however, to evaluate the protective potential of such vaccine candidates (171).

#### 6.2.5 Viral Vector-Based Vaccines

Antigens delivered in the form of genetic information will be expressed in the natural environment of the cell, which helps to ensure the correct antigen generation and processing that is vital for a potent immune response. Vectored vaccines deliver the genetic material encoding the antigen of interest by incorporation into unrelated live attenuated or replication incompetent viruses (**Figure 5B**). A large number of viral vectors are available that each differ in their ability to stimulate different arms of the immune system, but also in more practical characteristics including genomic stability, accepted insertion size, and safety profile (172).

Experimental NA-based vector vaccines have been described among others for pox virus (173, 174) or parainfluenza virus (175) vectors. Modified Vaccinia Ankara (MVA), a safe and commonly used live poxviral vector, that expressed NA (MVA-NA) of H1N1pdm09 was analyzed for its protective efficacy. The MVA-NA vaccine induced high NAI titers and a potent cellular response characterized by particularly strong activation of CD8 T cells. Immunization conferred partial protection against replication of a homologous virus (173). A raccoonpox vector expressing the NA of avian H5N1 conferred full protection to mice against an otherwise lethal challenge when administered *via* the intranasal (IN) route. Interestingly, depletion of CD4 and CD8 T cells strongly reduced protection, suggesting an important role for cellular immunity in the protection provided by this vector (174). In contrast, parainfluenza 5 (PIV5) expressing the NA of H1N1pdm09 or avian H5N1 induced weak T cell responses and was dependent on antibodies for protection. PIV5 expressing the H1N1pdm09 NA conferred partial cross-protection against H5N1 and H3N2 challenge, whereas PIV5 expressing the H5N1 NA protected against H1N1, but not H3N2 (175). Other NA-expressing viral vectors that have been applied with varied success in a veterinary setting are Newcastle disease virus, infectious laryngotracheitis virus and alphavirus replicons (176–179).

#### 6.2.6 Nucleic Acid-Based Vaccines

Nucleic acid-based vaccines directly deliver the genetic information encoding the antigen without the need for a viral vector (**Figure 5B**). In doing so, these methods exploit the benefits of *in vivo* antigen expression while eliminating the risks for reduced efficacy due to anti-vector immunity and safety concerns associated with viral vector-based vaccines. Production of this type of vaccines is rapid and scalable, which could be a critical advantage over other vaccine platforms in the event of a new emerging strain. Recent advances in stability and delivery of RNA and DNA vaccine formulations have led to improved immunogenicity, resulting in a renewed interest in using these technologies for emerging infectious diseases (172, 180, 181). mRNA vaccine technology was particularly accelerated by the current SARS-CoV-2 pandemic with the development of two highly effective vaccines based on this technology (182, 183).

Several studies reported the protective potential of NA antigens delivered by vaccination with plasmid DNA. An early study comparing the ability of various IAV antigens delivered by plasmid DNA to induce a protective immune response showed that only immunization with HA or NA, but not other internal IAV antigens, protected mice against homologous H1N1 challenge (184). In a follow-up study DNA vaccination with N2 induced full protection against challenge with homologous and drifted H3N2 strains, but was not effective in protecting against H1N1 challenge (185). Plasmid DNA encoding N1 from H1N1 conferred full protection against homologous challenge and 40-50% protection against H5N1 (186). Efficacy of a H5 DNA vaccine against challenge with a distant H5N1 strain was boosted from 75% to 100% with the addition of a N1-encoding plasmid (187).

NA-based mRNA vaccines have demonstrated high potency in some studies, but lower in another (188–190). Lipid encapsulated mRNA vaccines encoding various antigens of H1N1pdm09, separate or in a combination vaccine, elicited a strong humoral and cellular response in mice. The NA component of the vaccine was found to be the only one eliciting high NAI titers and protecting against a highly lethal dose of a matched challenge virus. A vaccine dose as low as 0.05 μg was sufficient to elicit a protective immune response. The NA mRNA vaccine however provided only limited or no protection against heterosubtypic challenge while the other more conserved vaccine components were fully protective (190) Similarly, in a recent study vaccination with mRNA encoding NA of H1N1pdm09 induced high NAI titers and protected against mortality from challenge with pre-pandemic H1N1 and H5N1. Serum antibodies from vaccinated mice however did not cross-react with H3N2 or influenza B virus (189) In an earlier study the immunogenicity of a N1-mRNA vaccine was considerably lower. High dose vaccination induced only 40% protection against a matched challenge in mice. Supplementing a H1-mRNA vaccine with the N1-mRNA however resulted in significantly reduced morbidity over the H1-only vaccine (188). The potential of mRNA vaccines might be boosted further by the use of self-replicating RNAs, which may induce high expression levels after low dose vaccination (191, 192).

## 7 Outlook

Various studies mentioned in this review describe NA-based vaccine candidates with impressive protective efficacy against homologous and heterologous challenge strains, although most candidates have yet to be tested in models other than mice. In view of the potential of NA to induce protective immunity, efforts to improve vaccine efficacy against influenza should not only focus on HA, but also on NA. Improving the NA component of current vaccines with respect to antigenic match and immunogenicity, would likely improve the efficacy of these vaccines in the short term. NA additionally should be considered in the development of next-generation vaccines, besides the largely HA-focused approaches.

While studying the protective efficacy of isolated NA-based vaccine candidates is informative, NA particularly has potential as a component in a multi-antigen vaccine. Vaccines targeting both HA and NA provide better and broader protection, as evidenced by reduced disease and transmission when compared to HA- or NA-only vaccines. Given that the antigenic drift of HA and NA is discordant (76) a seasonal vaccine combining both antigens would be less likely to be mismatched with circulating strains for both antigens, compared to a HA-focussed vaccine.

Most vaccine candidates described here induce a protective immune response against homologous and in some cases intra-subtypic heterologous NA, but not heterosubtypic NA. The application of strategies aimed at increasing the breadth of the immune response is vital to improve protection against drifted or new emerging strains. The induction of broadly protective heterologous immune responses may be enhanced by computational design of consensus antigens. Protective responses against different NA subtypes may be achieved by simple mixing of NA antigens, though the breadth may in such vaccines still be limited to the strains used for immunization like in the current seasonal vaccines. Additional measures may be required to ensure that the elicited immune response surpasses the immunization strains. To direct the immune responses more towards conserved epitopes the option of combining NAs from multiple strains onto heteromultivalent mosaic nanoparticles could be explored.

The recent market application of mRNA vaccines targeting SARS-CoV-2 is likely to pave the road for the clinical use of this vaccine platform for novel influenza vaccines. Prior to the emergence of SARS-CoV-2 it was already recognized that mRNA vaccines would be suitable specifically in an outbreak setting mostly due to the capacity for rapid development, in addition to the low dose requirement and high potency (180). mRNA vaccines encoding HA of potential pandemic strains have already demonstrated safety and immunogenicity in ferrets, non-human primates and humans (193) and clinical trials for seasonal HA-based mRNA vaccines are underway (Clinical Trials Identifiers NCT04956575 and NCT04969276). Addition of NA-encoding mRNA to such formulations is likely to improve the magnitude and breadth of protection and should be advocated.

## Author Contributions

SC, MP, MB, XS, and CH wrote the manuscript. SC, MP, and MB created the figures. MB made **Table 1**. All authors contributed to the article and approved the submitted version.

## Funding

This work was supported in part by the ENDFLU project that has received funding from the European Union’s Horizon 2020 research and innovation programme under grant agreement No 874650. The present work was also a part of the research program of the Netherlands Centre for One Health (www.ncoh.nl) and was financially supported through the One Health Investment Fund from the Faculty of Veterinary Medicine of the Utrecht University.

## Conflict of Interest

XS declares to receive funding from Sanofi Pasteur for research related to influenza vaccine development.

The remaining authors declare that the research was conducted in the absence of any commercial or financial relationships that could be construed as a potential conflict of interest.

## Publisher’s Note

All claims expressed in this article are solely those of the authors and do not necessarily represent those of their affiliated organizations, or those of the publisher, the editors and the reviewers. Any product that may be evaluated in this article, or claim that may be made by its manufacturer, is not guaranteed or endorsed by the publisher.
